# Effect of Replacing Soybean Meal by Raw or Extruded Pea Seeds on Growth Performance and Selected Physiological Parameters of the Ileum and Distal Colon of Pigs

**DOI:** 10.1371/journal.pone.0169467

**Published:** 2017-01-06

**Authors:** Anna Tuśnio, Marcin Taciak, Marcin Barszcz, Ewa Święch, Ilona Bachanek, Jacek Skomiał

**Affiliations:** Department of Monogastric Nutrition, The Kielanowski Institute of Animal Physiology and Nutrition, Polish Academy of Sciences, Jabłonna, Poland; Max Rubner-Institut, GERMANY

## Abstract

The use of pea seeds is limited due to the content of antinutritional factors that may affect gut physiology. Heat treatment such as extrusion may reduce heat-labile antinutritional factors and improve the nutritional value of pea seeds. This study determined the effect of partial replacement of soybean meal in pig diets by raw or extruded pea seeds on growth performance, nitrogen balance and physiology of the ileum and distal colon. The experiment was carried out in 18 castrated male piglets of initial body weight of 11 kg, divided into three groups. The animals were fed cereal-based diets with soybean meal (C), which was partly replaced by raw (PR) or extruded pea (PE) seeds. Nitrogen balance was measured at about 15 kg body weight. After 26 days of feeding, tissue samples were taken from the ileum and distal colon for histological measurements, and colonic digesta samples for analyses of microbial activity indices. The animals fed the PE diet had a significantly greater average daily gain than those fed the C diet and better apparent protein digestibility than those on the PR diet. Pigs fed the PR diet had a significantly greater butyric acid concentration and lower pH in the colon than pigs fed PE and C diets. There was no significant effect of the diet on other indices of microbial activity or morphological parameters. In conclusion, feeding a diet with extruded pea seeds improved growth performance of pigs, did not affect intestinal morphology and had a negligible effect on microbial activity in the distal colon.

## Introduction

Currently in Europe, protein requirements are mainly covered by soybean meal, which is widely used in diets for farm animals. Owing to the global rise of its price, it is necessary to search for alternative protein sources. It seems that legumes, e.g. lupin, faba bean and pea seeds are good alternative sources with a relatively high content of vegetable protein [1], and can be used both for animals and humans [2]. It is known that legumes contain lower level of sulphur amino acids and tryptophan than soybean meal [3, 4, 5], but the addition of crystalline amino acids to the diets for growing pigs or mixing them with cereals improve the protein value [6, 7, 8]. In terms of amino acid digestibility, pea is a better source of protein than lupins or beans [9, 10]. Pea seeds are also regarded as an important source of carbohydrates, vitamins and some minerals, including trace minerals [3]. The use of legumes is limited due to the content of some antinutritional substances. These compounds, i.e. phytic acid, condensed tannins, lectins, protease and α-amylase inhibitors, decline the nutritional value of pea [11, 2] and may affect gastrointestinal tract physiology [12]. For example, tannins, protease inhibitors and lectins reduce feed intake, decrease nutrient digestibility and impair animal performance [13, 14, 15]. Furthermore, tannins may cause detrimental effects on the chicken and rat intestinal mucosa, leading to atrophy and shortening of villi and distortion of their architecture [16]. Excessive consumption of α-galactosides, which are part of the carbohydrate fraction in some grain legumes [17], 1994, may also cause undesirable effects. These carbohydrates, are not hydrolysed by digestive enzymes of monogastric animals but are degraded by bacterial α-galactosidase in the colon [18] and may contribute to flatulence and diarrhea in animals. On the other hand, they are also potential prebiotics [19] that induce changes in microbiota activity, particularly resulting in bacterial production of short-chain fatty acids (SCFA) [20] in the large intestine [21]. Butyric acid is the most important one, because it is the preferred fuel for colonic epithelial cells [22] and affects cell differentiation [23].

There are many feed processing techniques that improve the nutritional quality of legumes, e.g. extrusion cooking, germination, dehulling and cooking [24, 25, 26, 27, 28, 29]. Extrusion cooking is a hydrothermal process leading to partial or total destruction of thermolabile antinutrients, such as trypsin inhibitors or lectins. Besides, it has a relatively high efficiency, requires shorter cooking time than other heating processes and may be used on a large scale.

The level of condensed tannins in pea seeds is associated with flower colour [30, 31] and 1% of these compounds is considered a high level in colour-flowered peas [32]. Recently, a considerable progress in plant breeding has been observed, and low-tannin legume cultivars have become available [33]. In the light of this tendency, there is a need to re-evaluate the nutritional and physiological characteristics of modern cultivars of pulses. Milwa pea is a new colour-flowered variety with low tannin content (< 0.1%).

The aim of the present study was to assess whether partial replacement of soybean meal by raw or extruded pea seeds of Milwa variety in diets for piglets had a negative impact on growth performance, intestinal morphology and microbial activity in the distal colon.

## Materials and Methods

### Material

Seeds of the spring pea cultivar (*Pisum sativum* L.) of colour-flowered Milwa variety were purchased from a commercial supplier. Part of the seeds was extruded on a KMZ-2 single-screw extruder (Sharkan, Russia) under the following conditions: moisture of about 22%, exposition time of 10 s, temperature 110–135°C and 30 kg/cm^2^ pressure. Soybean meal was purchased from a commercial production plant.

### Animals, diets and experimental design

Animal care protocols and experimental procedures were approved by the Third Local Animal Experimentation Ethics Committee (resolution number 53/2012) at the Warsaw University of Life Sciences-SGGW, Poland. Eighteen castrated male piglets (PIC x Penarlan P76) of initial body weight (BW) of 11 ± 0.5 kg, weaned at 28 days of age, were divided into three groups of six animals each. Piglets were fed cereal-based diets containing ca. 18% crude protein, with soybean meal (control group—C) or with soybean meal partly replaced by raw (PR) or extruded (PE) pea seeds. In PR and PE diets, 30% of soybean meal protein was replaced by pea seed protein. The composition of diets is listed in Table 1. Animals were housed individually in cages with free access to water. Feed was restricted and offered in equal portions three times a day. Feeding level amounted to about 5% of BW (90% *ad libitum* feed intake). The diets were formulated according to the Nutrient Requirements of Swine [34], Nutrient Requirements and Nutritional Value of Feeds for Swine [35] and amino acids requirements [36]. The nitrogen balance experiment was performed on piglets at about 15 kg BW and it consisted of 7 days of initial period and 6 days of collection of urine and faeces. Individual BW was recorded once a week and feed consumption was measured daily. After 26 days of feeding, the animals were stunned by electric shock, exsanguinated and the small and large intestine was dissected. Tissue samples from the ileum (ca. 50 cm length before the ileal-ceacal valve) and distal colon (ca. 50 cm before the anus) were taken for histological measurements, whereas colonic digesta samples were collected for analyses of pH, concentrations of SCFA and amines, bacterial enzyme activities, and relative amounts of selected bacterial populations.

**Table 1:** Composition of experimental diets, g/kg air dry basis.

Component	Peas		Diets		
	raw	extruded	C	PR	PE
Peas					
raw			-	181.0	-
extruded					163.5
Wheat			420.0	420.0	420.0
Barley			100.0	100.0	100.0
Soybean meal			263.0	184.5	184.5
Rapeseed oil			25.0	36.0	36.0
Limestone			10.0	12.0	12.0
Salt			3.5	3.5	3.5
Mono-Calcium-phosphate			15.0	15.0	15.0
Mineral-vitamin premix^1^			4.4	4.4	4.4
Maize starch			146.6	33.1	50.6
L-lysine HCl (78%)			11.0	8.5	8.5
L-threonine (98%)			1.0	0.5	0.5
DL-methionine (98%)			0.5	1.0	1.0
L-tryptophan (98%)			-	0.3	0.3
Analysed, g/kg DM					
Dry matter	899.9	928.1	912.3	912.8	916.8
Crude protein	262	233	197.9	210.9	210.6
Crude fat	17.6	12.1	2.62	3.63	3.66
Crude ash	30.7	30.4	4.88	5.10	5.56
Crude fiber	65.8	75.2	2.64	3.47	3.22
N-NDF			0.59	0.56	0.56
Starch	342	346	481	440	453
Tannins	0.94	1.27	0.626	0.777	0.809
TIA	2.35	0.35	0.076	0.264	0.145
Essential amino acid					
Lys			16.50	15.20	15.40
Thr			7.24	7.42	7.32
Met			3.05	3.48	3.52
Cys			2.81	3.14	2.93
Ile			7.02	7.58	7.93
Val			7.72	8.83	8.27
Leu			12.2	13.1	14.0
Phe			8.11	9.57	8.68
His			3.82	4.44	4.00
Arg			10.20	13.30	11.90
Gly			6.99	8.17	7.56
None-essential amino acid					
Tyr			5.29	6.26	5.73
Ala			6.86	7.75	7.53
Asp			15.1	17.5	17.7
Glu			39.1	42.7	41.7
Ser			8.50	9.03	9.66
Pro			11.60	11.80	12.90

C, control group; PR, diet with raw pea; PE, diet with extruded pea; 1, provided per kg diet, jm/kg: vit. A 200000; vit D_3_ 20000; mg/kg vit. E 600; vit K_3_ 20; vit. B1 30; vit. B_2_ 60; vit. B_3_ 300; pantotheic acid 12.24; vit. B_6_ 40; mcg/kg: vit B_12_ 400; biotin 1000; mg/kg: vit. C 800; folic acid 6; Fe 24; Mn 16; Cu 4; Zn 24; J 0.32; Co 0.16; Se 0.08; g/kg 0.094

### Chemical analysis

The chemical composition of pea seeds, diets and excreta was analysed using standard methods [37]. Amino acid composition in the diets was determined using Acquity UPLC System (Waters, Milford, MA, USA) with a photodiode array detector (PDA) and AccQ·Tag Ultra C-18 column (2.1 x 100mm; 1.7 μm) (Waters, Milford, MA, USA). The condensed tannin content in the raw and extruded pea seeds was determined colourmetrically according to the previously described method [38] with modifications [39]. Trypsin inhibitor activity (TIA) was also analysed and expressed as mg pure trypsin inhibited per gram of sample as described elsewhere [40].

### *In vitro* analysis

Ileal protein digestibility was evaluated using a 2-step incubation procedure at pH 2.0 for 6 h with porcine pepsin and at pH 6.8 for 18 h with pancreatin [41]. The standardized *in vitro* ileal digestibility of protein was calculated as described previously [41, 42].

### Histological analysis

Tissue samples of the ileum and distal part of the colon were placed in Bouin’s solution for 5 days, dehydrated and infiltrated with paraffin wax. The samples were cut on a microtome (Microm 350, Germany) into 5 μm sections and stained with haematoxylin and counter-stained with eosin. Villus height, crypt depth, width of tunica muscularis and tunica mucosa were measured in ileal sections, whereas crypt depth and tunica muscularis were analysed in colonic sections. These parameters were evaluated using a light microscope (Zeiss, Axio Star Plus type, Göttingen, Germany) and an Axio Vision LE Rel. 4.5 image analysis program (Carl Zeiss, 2002–2005). Averages for each parameter represent at least 20 measurements.

### Digesta pH and SCFA analysis

The colonic digesta was mixed with ultrapure water in a ratio of 1:2 and pH was measured using a digital pH-meter (WTW pH/340, Germany). Afterwards, 1 M NaOH solution was added to digesta samples to adjust the pH to at least 8.2, then the samples were centrifuged (10 min, 1800 *g*) and stored at -20°C for further analysis.

The SCFA analysis was carried out as described earlier [43] using an HP 5890 Series II gas chromatograph (Hewlett Packard, Waldbronn, Germany) with a flame-ionization detector. A Supelco Nukol fused silica capillary column (30 m x 0.25 mm internal diameter, film 0.25 mm) was applied and helium was used as the carrier gas. The concentrations of individual SCFA were estimated in relation to an internal standard (isocaproic acid) using a mixture of SCFA standard solutions.

### Amines analyses

Amines were analyzed as dansyl chloride derivatives with the HPLC method [44], using SEP-PAK C18 solid-phase extraction cartridges (6 ml, 500 mg Waters Ltd., Watford, Hertfordshire, UK). The separation was performed using a Finnigan Surveyor Plus liquid chromatograph (Thermo Scientific, San Jose, USA) with a photodiode array detector operated at 254 nm. The separation of amines was performed with a Waters Symmetry Shield RP_18_ column (150 x 3.9 mm i.d., particle size 5 μm). Heptylamine was used as an internal standard and amines were identified and quantitated based on the standard curves prepared for each amine.

### Bacterial DNA isolation and semiquantitative analysis of selected bacterial groups

Samples of colonic digesta for the isolation of bacterial DNA were treated according to the previously described method [45], and DNA was extracted using Wizard Genomic DNA purification kit (Promega, Madison, WI, USA) and the modified manufacturer’s protocol [46]. The concentration and purity of DNA were analysed using a NanoDrop ND-1000 spectrophotometer (Thermo-Scientific, Wilmington, DE, USA). The primers used to amplify bacterial 16S rRNA gene are presented in Table 2. Total bacterial population was evaluated using a universal primers set. PCR mixture contained: 5 μl of isolated DNA (50 ng), 25 μl of 2x PCR *TaqNova*-RED Master Mix (BLIRT S.A. DNA-Gdańsk Division, Gdańsk, Poland), 5 μl of each primer and 10 μl of nuclease-free water. The amplification of the 16S rRNA gene was performed for all bacteria (*Lactobacillus* spp., *Escherichia coli*, *Bifidobacterium* spp. and for *Clostridium* spp.) as described previously [47, 46]. PCR products were visualized by densitometric scanning with an ImageJ 1.47v programme (National Institute of Mental Health, Bethesda, MD, USA) after electrophoresis in a 2% agarose gel stained with ethidium bromide. The relative amounts of the analysed bacteria were calculated in relation to the density of the PCR product of the universal primers set and expressed in arbitrary units.

**Table 2:** Primers used for PCR amplification of bacterial 16S rRNA gene.

Bacteria	Primers	Sequence (5’-3’)	Product lengh [bp]	Reference
Total	Forward	CGTGCCAGCCGCGGTAATACG	611	[48]
	Reverse	GGGTTGCGCTCGTTGCGGGACTTAACCCAACAT		
*Lactobacillus* spp.	Forward	CATCCAGTGCAAACCTAAGAG	286	[49]
	Reverse	GATCCGCTTGCCTTCGCA		
*Bifidobacterium* spp.	Forward	CGGGTGCTICCCACTTTCATG	1417	[50]
	Reverse	GATTCTGGCTCAGGATGAACG		
*Clostridium* spp.	Forward	AAAGGAAGATTAATACCGCATAA	722	[48]
	Reverse	ATCTTGCGACCGTACTCCCC		
*Escherichia coli*	Forward	GGGAGTAAAGTTAATACCTTTGCTC	585	[51]
	Reverse	TTCCCGAAGGCACATTCT		

### Bacterial enzymes activity

The activity of β-glucuronidase and β-glucosidase in the colonic digesta was measured colourimetrically [43, 46]. Phenolphthalein β-D-glucuronide (75 mM) was the substrate for β-glucuronidase and 5 mM *p*-nitrophenyl-β-D-glucopyranoside was the substrate for β-glucosidase. The absorbance was measured at 540 and 400 nm, respectively, using a Unicam UV300 spectrophotometer (Thermo-Spectronic, Cambrige, UK).

### Statistical analysis

The results were subjected to one-way analysis of variance and the differences were evaluated with the post-hoc Tukey’s HSD test using the STATGRAPHICS^®^ Centurion XVI ver.16.1.03 statistical package (StatPoint Technologies, Inc., Warrenton, Virginia, USA). The effects were considered to be significant at P ≤ 0.05.

## Results

The chemical composition of raw and extruded pea seeds is shown in Table 1. The dry matter (DM) content of raw seeds was lower in comparison with the extruded pea (899.9 *vs*. 928.1 g/kg, respectively). Both peas were similar in crude protein (262 and 233 g/kg of DM, respectively), crude ash (30.7 and 30.4 g/kg of DM, respectively), crude fibre and starch contents. Condensed tannin content was similar in both pea sources (0.94 and 1.27 g/kg of DM), whereas TIA was greater in raw than in the extruded pea and amounted to 2.35 and 0.35 mg/g, respectively.

The average daily gain and feed conversion ratio were higher in the PE group than in the C group (P = 0.025 and 0.006, respectively) (Table 3, S1 Appendix). The apparent protein digestibility in the whole gastrointestinal tract was significantly higher in PE and C groups as compared to PE (P = 0.026). N retention to N absorbed depended on the protein and was higher in the PE group when compared to the C group (P = 0.006). A similar dependence was observed in case of N retain to N intake (P = 0.017). The results of *in vitro* analysis of standardized ileal digestibility were consistent with the results of *in vivo* analysis and were significantly higher in the PE group than in PE and C groups (P = 0.005).

**Table 3:** Average daily gain and feed intake (kg), feed/gain ratio, apparent protein digestibility (%) in whole gastrointestinal tract, protein utilization (%) of piglets and *in vitro* standardized ileal digestibility (%) of experimental diets.

Item	C	PR	PE	SEM	P-value
ADG	0.502^a^	0.534^a^^b^	0.548^b^	0.007	0.025
Feed intake	20.5	20.9	20.6	0.181	0.702
F/G	1.57^b^	1.50^a^^b^	1.45^a^	0.018	0.006
Apparent protein digestibility	87.0^a^^b^	83.9^a^	87.2^b^	0.590	0.026
N retention/N absorbed	72.5^a^	77.0^a^^b^	79.0^b^	0.937	0.006
N retention/N intake	63.1^a^	64.6^a^^b^	68.9^b^	0.913	0.017
*In vitro*					
Standardized ileal digestibility	92.0^a^^b^	90.3^a^	93.1^b^	0.380	0.005

^a, b^, means with different letters differ significantly (P ≤ 0.05)

Partial replacement of soybean meal with raw or extruded pea seeds did not change morphological parameters of the ileum and colon (Table 4, S1 and S2 Appendices).

**Table 4:** Morphological parameters (μm) of the ileum and distal colon of pigs.

	C	PR	PE	SEM	P-value
	Morphological parameters				
Ileum					
tunica mucosa	496	508	476	12.34	0.595
villus height	296	286	268	6.59	0.219
crypt depth	225	213	209	6.46	0.617
tunica muscularis	808	760	763	26.43	0.736
villus/crypt ratio	1.43	1.45	1.39	0.04	0.856
Distal colon					
crypt depth	559	543	586	18.39	0.663
tunica muscularis	619	534	585	34.37	0.622

C, control group; PR, diet with raw pea; PE, diet with extruded pea;

Feeding PR and PE diets did not influence total SCFA, as well as acetic, propionic, isovaleric and valeric acid concentrations (Table 5, S4 Appendix). However, pigs fed the PR diet had a significantly greater butyric acid concentration (P = 0.013) and substantially lower digesta pH (P = 0.044) than pigs fed PE diet. There was also observed a tendency towards greater isobutyric acid concentration in colonic digesta of pigs fed PE than PR and C diets (P = 0.069).

**Table 5:** Digesta pH, SCFA concentration (μmol/g digesta) and bacterial enzymes activity (U/g digesta) in the distal colon of pigs.

	C	PR	PE	SEM	P-value
	pH and SCFA concentration				
pH	6.8^ab^^1^	6.6^a^	7.0^b^	0.07	0.044
Total SCFA	55.2	56.9	50.7	2.48	0.602
Acetic acid	32.1	30.0	29.3	1.26	0.682
Propionic acid	11.3	12.3	10.7	0.71	0.669
Isobutyric acid	1.1	1.0	1.4	0.06	0.069
Butyric acid	7.9^ab^	10.9^b^	6.1^a^	0.71	0.013
Isovaleric acid	1.5	1.4	1.9	0.12	0.184
Valeric acid	1.3	1.3	1.3	0.12	0.998
	Bacterial enzymes activity				
β-glucuronidase	30.2	16.5	25.6	3.41	0.263
β-glucosidase	89.3	76.7	91.5	8.47	0.767

C, control group; PR, diet with raw pea; PE, diet with extruded pea; SCFA, short-chain fatty acids;

^1^, within raws means with different superscripts are significantly different at P≤0.05.

No significant differences in bacterial enzyme activities (β-glucuronidase and β-glucosidase) were observed regardless of the treatment (Table 5, S5 Appendix).

**Fig 1:**
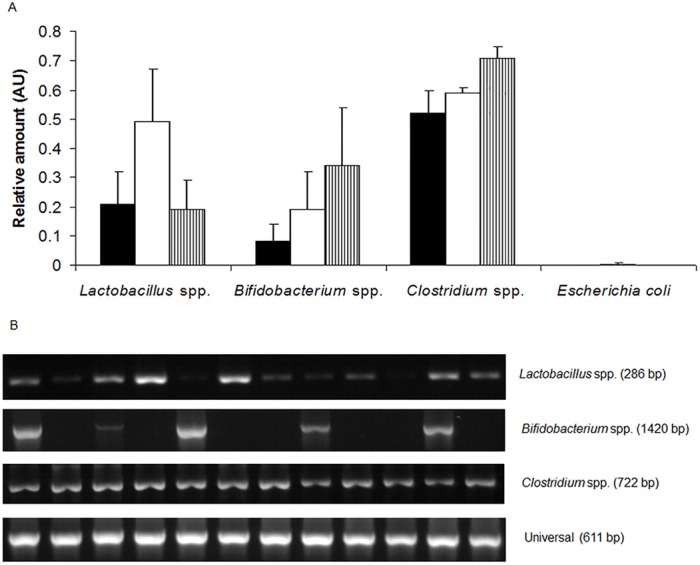
A. Relative amounts (arbitrary units, AU) of selected bacterial populations in the distal colonic digesta of pigs fed control (black bars), raw pea (white bars) and extruded pea (white bars with vertical lines) diets. Error bars represent standard error of the mean. Significance of effects in one-way ANOVA was as follows: for *Lactoabcillus* spp. (P = 0.266), for *Bifidobacterium* spp. (P = 0.461), for *Clostridium* spp. (P = 0.060) and for *Escherichia coli* (P = 0.121. B. Representative PCR products of 16S rRNA gene of *Lactobacillus*, *Bifidobacterium* and *Clostridium* species and invariant sequences of 16S rRNA gene of all known intestinal bacteria (Universal) in the distal colon of pigs. PCR products were placed randomly on agarose gels to avoid the possible position effect during electrophoresis.

Relative amounts of selected bacterial populations in the distal colon were not affected by the treatment (Fig 1, S6 Appendix). Only a tendency was observed towards an increase of *Clostridium* spp. populations when pigs were fed the PE diet in comparison with C and PR diets (P = 0.060).

The total concentration of amines was not affected by raw or extruded pea seeds in comparison with the C diet (Table 6, S7 Appendix). The effect of partial replacement of soybean meal by raw or extruded pea was very small. There was only a trend towards greater concentration of 1.7-diaminoheptane and spermidine in PE group compared to C and PR groups. The concentration of these amines was 0.055, 0.045 and 0.040 μmol/g digesta and 0.023, 0.015 and 0.017 μmol/g digesta, respectively.

**Table 6:** Concentration of amines (μmol/g digesta) in the distal colon of pigs.

	C	PR	PE	SEM	P-value
Spermidine	0.0152	0.0166	0.0229	0.00157	0.0973
Methylamine	0.0174	0.0163	0.0174	0.00045	0.5266
Putrescine	0.0317	0.0323	0.0274	0.00292	0.7755
Phenylethylamine	0.0080	0.0087	0.0093	0.00043	0.4415
Cadaverine	0.0263	0.0276	0.0392	0.00814	0.7973
Histamine	0.0196	0.0188	0.0209	0.00046	0.1775
1,7-Diaminoheptane	0.0446	0.0401	0.0548	0.00265	0.0563
Tyramine	0.0079	0.0050	0.0085	0.00106	0.3808
Tryptamine	0.0164	0.0114	0.0200	0.00171	0.1171
Total amines	0.1872	0.1752	0.2204	0.01637	0.1642

C, control group; PR, diet with raw pea; PE, diet with extruded pea;

## Discussion

Milwa pea represents new colour-flowering cultivar and is used as a feed ingredient mainly for monogastric animals. The colour of pea flowers, according to a previous study [52], is related to tannin content in seeds, which are recognized as the main antinutrient in colour-flowered pulses. The high tannin content (7.26–12.11 g/kg) in peas may decrease the digestibility of protein and metabolizable energy in rats and chickens [52]. In the present study, thermal process did not change their content but it is suggested that modern cultivars of peas, such as Milwa with a low tannin content (0.96–1.10 g/kg), can be accepted in feeds for broilers [53].

Trypsin inhibitor is another important antinutritional factor in pea is that can have a major impact on the nutritional value as it inhibits pancreatic serine proteases and stimulates pancreatic juice secretion, thereby reducing protein digestion. It leads to pancreatic hypertrophy in chickens [54] and poor growth performance in animals [55]. Trypsin inhibitors in pea seeds are similar in structure to Bowman-Birk inhibitors in soybean. Many researchers [56, 57, 58] reported that TIA in spring pea was within the range of 0.9–7.9 mg/g and Milwa pea, used in our study, also fell within this range. Heat treatment decreased TIA activity very effectively. Growth performance in the present study was significantly affected by dietary treatment and the extrusion process probably improved the protein value of pea seeds, which contributed to the increased protein digestibility in pigs fed the PE diet. Pigs fed the PE diet had greater average daily gain than pigs in the C group. Previously, it was shown that TIA concentration in the diet up to about 0.5 mg/g did not decrease nutrient digestibility in pigs [15]. In the present study both TIA and tannins were at low levels in the experimental diets (0.076–0.264 mg/g and 0.626–0.809 g/kg, respectively) and probably did not influence this parameter. Thus, the increased protein digestibility of the PE diet resulted probably from the exposure to moist heat treatment, under which the physical structure usually changes causing substantial differences in the reactivity as well as functional and nutritional properties [59]. Standardized ileal digestibility of protein estimated *in vitro* was consistent with the results *in vivo*. Literature data indicate a positive effect of extrusion on *in vitro* protein digestibility [60, 61, 62].

Modifications of the structure of intestine morphology are used to evaluate gut function in response to feed components. Some dietary components, e.g. antinutritional factors can affect intestinal morphology and its function [63]. It was shown that tannins did not induce morphological changes in the intestinal mucosa in pigs [64]. In the present study, the form of pea seeds (raw or extruded) had a negligible effect on the morphology of the ileum and distal colon. Similar results were observed previously [53] and indicated that pea seeds (raw or extruded) had no effect on the epithelium of chickens’ intestine.

Previous research has shown that legumes are a good source of dietary fibre that can be fermented in the colon by microbiota. Bifidobacteria and lactobacilli are considered as beneficial intestinal bacteria while *Escherichia coli* and *Clostridium perfringens* are undesirable for human and animals health [65]. Fermentation leads to the production of SCFA, such as acetate, propionate and butyrate [66]. In the present study, the total SCFA concentration in the colonic digesta was similar in all experimental groups. Partial replacement of soybean meal by raw or extruded pea did not significantly influence bacterial fermentation in the distal colon. The most important modifications concerned the concentration of butyric acid, which enhances cell differentiation, preventing colon pathogenesis [67]. In the current study, butyrate concentration was the highest in pigs fed diets with raw pea seeds, which was in agreement with the previous results [68], and significantly lowered pH of the distal colon in the same group. Literature data indicate [69, 70] that lower pH changes gut microbiota composition and prevents overgrowth of pathogenic bacteria, e.g. *Enterobacteriaceae* and clostridia. Contrary to expectations, feeding the PE diet resulted in a tendency of increasing *Clostridium* spp. populations in the distal colon of pigs. Insoluble fibre, which is present in legumes, is slowly fermented in the large intestine and may reduce proteolytic fermentation [71] and promote beneficial alteration in the microbial colonization with a higher butyric acid production in the large intestine and lower *enterobacteria* count in the digesta [72]. The results of our study also indicated a tendency towards greater isobutyric acid concentration in the colon of animals fed the PE diet. Branched-chain fatty acids (isobutyrate and isovalerate) are produced during proteolytic fermentation and are formed from branched-chain amino acids (valine, leucine and isoleucine) [73], therefore, slightly greater isobutyrate concentration is consistent with a trend of increased relative amount of *Clostridium* spp., which include many proteolytic species Changes in isoacids concentration may also be related to protein digestibility, but feeding the extruded pea should reduce the amount of protein reaching the large intestine that can be utilized by the microbiota. Improvement of protein quality is the main effect of heat treatment on the nutritional value of legume seeds, and heated legumes are better digested than the raw ones [74], but it is also possible that during extrusion, some portion of pea seed protein becomes resistant to digestion in the small intestine and reach the colon.

The colonic epithelium is exposed to many different toxic substances that can lead to its damage. These components include both endogenous factors produced within the organism and those delivered in the food [75]. Two enzymes, β-glucuronidase and β-glucosidase, are associated with carcinogenesis in the colon. These enzymes are involved in toxin deconjugation and their lower activity may lead to a reduced exposure to carcinogens [76]. Protein fermentation also leads to the production of potentially toxic substances, e.g. ammonia, amines, *N*-nitroso compounds, phenols and indoles [77, 78]. They may be toxic for intestinal enterocytes, impair barrier function and allow translocation of pathogenic bacteria [73, 79]. Amines are formed by decarboxylation of amino acids during active growth of some bacterial species e.g. C*lostridium perfringens* and *Bacteroides fragilis* [80]. Production of amines is probably closely related to the carbohydrates present in the digesta, and their lower availability leads to the utilization of protein as an energy source [44]. The total amine concentration in our study was not affected by dietary treatment. Only a tendency was observed to increased concentrations of 1.7-diaminoheptane and spermidine in pigs fed PE diet in comparison with C and PR diets. This trend was consistent with our results concerning isobutyric acid concentration, *Clostridium* spp. amount, and could indicate more intensive proteolysis. However, further studies are required to unravel the effect of extruded feed components on microbiota activity.

## Conclusion

Partial replacement of soybean meal with extruded pea seeds in the diet for pigs improved both the average daily gain and apparent protein digestibility. Feeding diets containing raw pea seeds exerted more beneficial effect on microbiota activity than extruded seeds, as it increased butyric acid concentration and did not contribute to the intensification of proteolysis in the distal colon of pigs. However, microbial activity and intestinal morphology did not considerably change due to the partial replacement of soybean meal with pea seeds, indicating that they were a good alternative protein source to soybean meal in diets for pigs.

## Supporting Information

S1 Appendix Growth performance. Raw Data.
(PDF)

S2 Appendix Histology of the ileum. Raw data.
(PDF)

S3 Appendix Histology of the distal colon. Raw data.
(PDF)

S4 Appendix pH and short chain fatty acids concentration. Raw data.
(PDF)

S5 Appendix Bacterial enzymes activity. Raw data.
(PDF)

S6 Appendix PCR. Raw data.
(PDF)

S7 Appendix Amines concentration. Raw data.
(PDF)
