# Supplementary material for: Effect of Replacing Soybean Meal by Raw or Extruded Pea Seeds on Growth Performance and Selected Physiological Parameters of the Ileum and Distal Colon of Pigs
Source: PLoS One. 2017 Jan 6;12(1):e0169467. doi: 10.1371/journal.pone.0169467 (PMC5218572; doi:10.1371/journal.pone.0169467)
Supplement: S1 Appendix — Raw Data. (PDF) [file pone.0169467.s001.pdf]

S1 Appendix. Growth performance, raw data.

	AID	N retention/N absorbed	N retention/N intake	SID in vitro	ADG
C	84,61164	74,73883956	63,23775819	92,06	0,463462
C	86,58244	74,86788774	64,82244083	92,21	0,517308
C	89,75677	77,51664659	69,57643515	92,1	0,548077
C	88,30144	66,56327008	58,77632337	91,79	0,465385
C	86,32767	67,22064757	58,03002009		0,480769
C	86,48882	74,38927305	64,33840093		0,536538
PR	86,14552	79,7575974	68,70759778	88,7	0,563462
PR	86,67128	75,97732477	65,8505201	91,45	0,515385
PR	85,44137	76,11915232	65,03724559	90,76	0,55
PR	83,34173	77,90999674	64,93153721		0,526923
PR	79,28092	75,89111685	60,16717381		0,530769
PR	82,55056	76,4892538	63,14230542		0,517308
PE	85,61299	79,98323829	68,47603874	92,76	0,567308
PE	86,01324	77,13800827	66,34890275	92,96	0,521154
PE	87,87541	81,81778906	71,89771746	93,72	0,575
PE	88,32883	78,63766584	69,45973267	92,85	0,548077
PE	86,09595	80,38068789	69,23224028		0,536538
PE	89,22533	76,09116589	67,89259346		0,542308

F/G	Feed intake
1,56805	18,895
1,517844	20,415
1,502807	21,415
1,68719	20,415
1,6332	20,415
1,535125	21,415
1,461638	21,413
1,493284	20,01
1,489231	21,296
1,563139	21,415
1,548986	21,376
1,469591	19,766
1,384068	20,415
1,506642	20,415
1,431237	21,397
1,432632	20,415
1,408459	19,648
1,518794	21,415
