# Supplementary material for: Effect of Replacing Soybean Meal by Raw or Extruded Pea Seeds on Growth Performance and Selected Physiological Parameters of the Ileum and Distal Colon of Pigs
Source: PLoS One. 2017 Jan 6;12(1):e0169467. doi: 10.1371/journal.pone.0169467 (PMC5218572; doi:10.1371/journal.pone.0169467)
Supplement: S2 Appendix — Raw data. (PDF) [file pone.0169467.s002.pdf]

S2 Appendix. Histology of the ileum, raw data.

No	Mucosa	Villi	C<br>Crypt	Muscularis
1	466,87	329,18	188,49	692,31
	432,03	288,88	288,18	708,97
	468,16	339,45	240,27	702,59
	375,11	270,02	206,38	682,13
	488,33	319,36	220,68	696,64
	467,13	301,92	152,74	693,34
	415,1	304,97	148,65	683
	372,05	340,74	204,52	705,85
	355,45	264,32	237,51	685,91
	561,77	241,05	222,33	679,54
	479,73	209,07	167,11	685,67
	493,53	305,23	254,14	697,48
	421,57	230,8	229,28	690,55
	452,54	184,09	178,56	935,44
	410,29	296,93	172,96	933,23
	389,54	275,11	150,71	946,2
	344,6	273,72	115,56	991,62
	361,84	254,74	222,75	1020,9
	398,49	241,79	211,01	987,9
	330,4	218,73	154,39	922,35
	439,47	319,05	165,03	911,99
	400,87	265,5	130,06	937,85
	516,04	214	205,94	923,77
	468,43	240,17	217,81	928,45
	480,75	235,85	167,96	926,78
	475,75	239,49	178,9	960,1
	469,68	176,25	139,83	696,36
	439,78	288,48	151,31	975,64
	396,64	289,57	191,43	920,96
	468,27	337,81	177,32	865,98
	428,17	311,99		826,16
	411,68	294,35		799,15
	435,65	268,44		756,32
	447,72	238,07		
	423,66	216,27		
	363,44	258,58		
	381,66	355,66		
	360,22	348,44		
	408,73	288,77		
	421,19	338,28		
	427,08	298,95		
1	428,0344	275,9529	189,727	823,36758
2	641,23	392,33	261,6	1020,84
	718,38	409,33	163,33	1018,44
	726,38	373,67	181,43	917,07

	665,86	334,58	286,32	846,8
	578	297,54	304,42	814,38
	451,33	329,22	253,66	779,11
	472,64	316,53	363,18	764,52
	550,2	298,56	330,7	769,89
	526,64	348,02	336,95	975,05
	474,96	273,83	289,31	806,06
	510,6	251,94	227,25	824,17
	651,09	343,29	255,58	838,18
	600,18	381,82	182,31	841,39
	563	426,25	187,98	798,98
	728,92	415,11	349,34	807,58
	710,92	378,39	274,56	981,32
	526,97	517,04	407,84	991,91
	624,83	442,37	291,42	1032,51
	562,04	326,25	231,41	1025,96
	566,73	297,7	335,72	1000,8
	608,58	340,61	430,98	1012,6
	558,1	432,34	362,37	1032,87
	662,02	371,93	325,62	1019,11
	589,73	422,58	255,45	722,62
	500,75	405,25	305,23	722,81
	635,54	407,77	351	740,5
	553,29	340,69	272,77	741,17
	551,81	302,44	363,35	736,93
	467,43	339,1	369,28	725,67
	541,6	350,59	278,61	701,54
	557,94	274,37	211,03	688,7
	579,78	341,55	133,13	697,87
	615,52	317,71		741,38
	516,75	212,41		770,59
	606,05	282,22		771
	467,48	260,45		815,98
	644,84	356,53		849,26
	615,7	368,12		887,5
	600,67	462,89		
2	582,679	352,3928	286,6603	848,23842
3	567,55	197,19	244,6	700,01
	615,8	313,61	211,67	677,22
	595,89	284	204,34	685,57
	520,68	258,77	259,97	712,42
	552,06	277,9	249,78	749,12
	452,76	333,67	257,85	780,47
	402,03	346,38	251,56	783,73
	428,97	318,92	267,08	829,06
	499,2	243,05	250,86	843,89
	435,26	345,58	205,09	792,43
	579,06	344,13	234,49	775,05

	598,39	303,99	246,89	643,76
	590,36	464,74	314,49	1096,58
	491,17	341,62	200,61	1050,47
	480,42	288,68	209,2	1037,5
	420,85	247,53	200,06	1029,46
	428,08	350,8	144,88	1004,28
	505,37	294,2	178,42	1006,21
	437,42	312,52	170,08	990,65
	511,11	259,87	217,76	1031,63
	639,42	250,24	235,33	1020,15
	652,92	287,6	221,22	1014,08
	538,93	282,78	325,83	1028,56
	555,11	278,74	274,54	1030,27
	645,48	413,1	240,23	1070,08
	476,46	434,36	222,39	1034,48
	498,26	265,8	148,1	1069,5
	644,5	231,77	312,25	1107,53
	675,7	350,63	240,44	1128,24
	579,37	302,7	151,58	1133,88
	566,99	227,55		1164,25
	580,77	334,3		1115,59
	451,06	182,78		1073,21
	457,46	300,82		1076,69
	520,35	403,56		1054,23
	428,82	341,87		995,59
	360,77	398,81		
3	523,9135	308,5016	229,7197	953,77333
4	511,48	347,59	239,66	377,7
	557,43	219,53	330,69	362,66
	518,74	277,46	319,26	362,6
	471,51	332,8	271,98	354,28
	580,59	311,3	208,66	365,03
	580,33	300,84	257,85	373,06
	509,83	294,59	254,47	393,01
	504,75	264,12	279,37	394,26
	483,82	223,76	271,54	388,34
	498,79	306,07	185,4	425,1
	418,22	260,2	301,84	420,92
	419,73	311,88	299,42	399,38
	513,53	322,17	167,01	393,79
	572,4	223,03	190,58	425,34
	604,45	205,73	166,74	440,25
	608,76	267,42	235,4	476,69
	477,27	225,29	267,97	501,21
	481,78	214,06	156,76	501,31
	390,51	306,09	304,95	501,67
	491,71	232,6	309,17	531,66
	455,65	204,18	259,77	553,25

	467,88	182,36	283,74	557,15
	425,43	239,22	266,19	580,46
	388,67	308,84	278,29	606,43
	376,95	238,13	211,5	1054,04
	437,22	248,69	249,68	989,2
	365,47	297,25	190,13	1041,36
	397,41	255,95	166,15	1029,83
	533,86	310,91	236,7	995,69
	450,34	305,01	184,31	1003,93
	442,66		221,86	994,9
			220,99	949,06
				871,77
				913,76
				916,43
				975,09
				1026,77
4	481,8442	267,9023	243,3759	633,71297
5	587,53	221,23	154,06	898,24
	509,7	233,7	154,43	882,23
	470,77	217,03	245,08	880,26
	515,73	225,13	166,18	883,88
	376,95	271,62	177,35	904,46
	556,69	287,46	304,08	879,97
	394,97	342,54	226,4	826,75
	310,01	268,84	255,24	842,08
	304,38	273,04	183,74	867,94
	439,71	317,62	308,16	861,78
	450,69	426,37	166,07	845,67
	375,47	333,62	136,86	883,77
	557,51	359,42	156,55	893,45
	539,08	324,95	117,57	905,05
	553,68	381,53	112,54	910,99
	478,18	346,33	206,42	903,93
	471,67	369,84	224,62	896,64
	473,31	274,73	171,72	869,85
	596,88	267,35	163,65	856,45
	591,77	205,76	198,93	845,87
	641,3	247,25	119,52	855,77
	524,3	410,92	101,66	862,67
	477,35	344,06	144,77	842,27
	475,12	401,96	126,27	838,37
	513,47	331,12	112,5	822,96
	490,1	313,36	104,16	842,96
	517,29	335,24	153,92	823,81
	554,59	444,92	170,13	813,21
	524,33	399,49	281,24	839,76
	543,16	292,74	181,56	853,88
	593,43	248,21	215,4	882,04

	426,85	360,44	176,41	899,01
	309,73	228,34	153,23	909
	442,55	285,93	185,61	
	472,64	311,34	143,16	
			128,43	
			102,52	
			143,64	
			330,75	
			186,18	
			126,2	
			200,68	
			142,45	
			283,9	
			164,38	
5	487,454	311,5266	177,9627	867,42333
6	356,89	274,5	201,33	678,42
	371,37	299,91	152,75	662,69
	492,53	194,76	186,95	676,55
	441,09	210,71	280,9	583,49
	496,03	269,45	197,25	605,91
	431,49	245,73	202,99	544,03
	439,96	234,85	190,39	564,92
	437,59	268,2	172,72	596,33
	426,02	204,9	220,93	604,36
	498,61	273,85	271,26	614,32
	518,58	403,47	223,02	599,09
	412,49	273,16	185,83	578,13
	489,05	213,31	274,84	576,32
	547,59	268,91	274,95	564,24
	420,63	208,6	278,24	759,68
	528,05	240,27	232,77	785,93
	488,92	252,73	165,8	787,08
	525,2	198,26	206,27	776,47
	493,05	302,31	177,12	832,16
	374,41	238,63	208,74	802,39
	565,89	325,17	294,85	793,66
	456,98	253,75	340,47	817,49
	521,17	308,5	212,41	815
	516,34	328,63	148,22	811,38
	538,27	263,82	137,88	843,22
	538,26	250,48	273,21	825,65
	498,15	235,4	185,41	807,11
	466,82	270,04	285,71	810,47
	512,07	271,43	208,04	789,79
	381,79	273,03	213,71	779,1
			249,8	787,83
			283,43	796,55
			247,08	811,55

				778,35
6	472,843	261,892	223,7961	719,40176

No			PR	
	Mucosa	Villi	Crypt	Muscularis
13	594,79	308,47	297,14	961,51
	657,57	373,83	134,83	975,64
	559,77	341,37	114,94	1037,95
	462,25	257,91	249,59	981,83
	679,07	294,7	254,95	995,66
	443,93	318,77	279,47	1021,65
	521,07	309,28	294,91	989,48
	757,27	382,74	195,68	959,66
	685,59	311,94	277,89	999,4
	483,13	278,36	228,89	817,19
	533,73	184,1	194,45	785,26
	470,98	381,97	341,54	783,98
	383,63	338,72	266,38	794,21
	469,65	361,27	308,02	783,84
	442,3	274,63	302,28	780,2
	462,9	282,97	170,73	800,79
	642,46	215,02	278,79	817,69
	451,48	304,7	265,97	866,39
	652,14	247,74	200,86	883,6
	618,66	286,32	236,93	914,08
	510,47	360,54	140,35	933,15
	510,47	294,22	112,19	965,59
	409,97	275,8	180,21	978,84
	589,09	527,04	230,57	818,42
	442,74	347,45	266,29	799,02
	656,99	353,02	220,57	764,9
	532,34	255,05	133,87	747,47
	570,68	267,27	147,32	786,7
	548,77	271,62	243,95	785,54
	493,75	262,91	212,58	791,55
	469,16	221,54	255,67	813,95
	570,06	290,85	234,41	791,62
	778,01		237,63	810,79
			258,22	804,66
			180,86	780,38
			173,11	801,23
				810,68
				814,74
13	547,1173	305,6913	225,6122	861,8221
14	589,96	293,38	365,76	928,24
	596,91	368,72	343,94	945,08
	784,12	276,86	383,69	958,15
	499,71	432,82	192,23	957,94
	670,03	300,91	311,67	876,97
	607,33	241,46	314,68	897,92

	503,47	306,57	214,04	929,01
	594,93	338,29	286,71	921,83
	778,37	487,46	239,48	902,68
	718,72	454,68	248,37	956,96
	772,5	434,37	240,11	896,44
	685,57	336,61	286,69	916,75
	562,71	295,41	235,75	907,63
	487,51	341,78	181,98	952,11
	632,42	320,18	194,13	949,83
	508,25	264,45	210,11	940,78
	512,98	394,64	197,5	960,59
	546,99	224,13	189,52	912,26
	443,03	288,28	253,27	859,52
	574,44	307,25	401,05	857,08
	582,72	321,12	126,24	875,28
	615,64	349,65	300,65	855,07
	648,1	321,8	245,81	861,89
	479,53	250,38	226,44	865,29
	491,92	360,2	240,69	870,26
	611,74	325,39	219,4	889,09
	484,91	285,42	235,8	879,92
	679,48	288,16	232,02	868,82
	796,58	218,39	319,04	896,16
	618,45	295,2	271,3	897,66
	550,22	298,69	173,11	886,98
	425,61	416,63	191,65	883,85
	493,73	336,24	151,73	875,55
			380,22	883,51
			208,11	
			145,94	
			162,43	
14	592,3812	326,5309	246,5205	903,4441
15	559,02	305,74	280,73	692
	545,47	342,47	160,77	681,13
	506,41	279,29	160,78	704,86
	439,75	210,59	197,74	673
	405,03	277,9	177,65	818,72
	419,81	275,01	106,38	816,4
	367,36	277,43	148,73	757,74
	427,43	284,36	129,29	762,73
	536,09	289,16	233,88	749,25
	435,78	354,25	319,35	765,18
	388,24	343,75	159,91	762,89
	397,61	270,93	154,22	742,56
	444,42	187,17	180,94	731,52
	395,48	249,98	215,42	744,12
	366,99	236,73	239,06	764,01
	375,11	255,58	279,53	707,38

	388,87	211,7	252,43	739,56
	392,18	216,67	228,99	552,42
	357,39	226,08	160,73	559,44
	310,63	227,55	151,88	559,44
	319,19	218,63	177,87	557,43
	377,69	204,38	152,36	541,28
	416,12	258,81	180,52	555,49
	503,47	278,95	291,92	576,61
	550,29	350,82	138,41	560,33
	411,24	157,83	154,64	549,3
	462,92	220,97	179,35	580,56
	403,3	260,7	160,33	573,91
	423,5	225,12	252,98	568,78
	414,81	230,84	184,53	568,91
	394,1	317,17		575,22
	413,57	270,65		557,77
	444,11	297,31		577,47
	460,23	269,98		622,92
	432,23	275,58		635,98
	435,96	287,31		689,01
	485,98	326,69		673,04
	432,24	290,46		691,08
	393,39	188,4		
15	426,4977	262,8959	193,7107	656,3011
16	594,14	352,26	270,26	855,4
	637,56	366,83	228,18	830,51
	550,58	271,85	172,96	882,29
	519,23	265,57	133,31	857,81
	448,52	236,17	164,25	860,2
	605,83	198,95	241,09	858,74
	442,53	194,9	312,97	869,24
	405,28	240,7	170,61	868,85
	606,02	255,67	299,56	843,04
	656,43	325,69	207,85	873,18
	496,41	343,51	207,9	825,73
	540,97	300,92	230,16	827,27
	494,99	257,12	189,06	823,03
	430,51	295,84	168,03	814,18
	466,9	241,78	187,64	805,69
	426,72	201,89	125,48	746,75
	325,07	237,92	119,13	766,48
	425,58	227,38	253,88	717,65
	443,07	272,23	161,57	697,65
	430,48	283,84	179,58	690,19
	379,04	246,19	254,07	694,39
	489,23	204,37	228,89	695,4
	464,85	266,74	119,47	702,27
	503,5	291,66	95,12	714,79

	476,27	351,02	169,75	710,07
	429,16	342,12	162,53	714,38
	532,98	246,97	113,72	736,3
	355,25	262,09	179,78	754,34
	509,52	337,87	142,01	747,35
	478,07	233,55	220,09	714,3
	447,46	315,72	146,29	695,72
	521,5	300,49		708,38
	459,71	266,79		695,01
	429,34	258,72		694,33
	498,54			690,47
				680,01
				673,01
				659,58
16	483,464	273,3918	188,8771	762,9995
17	620,22	274,1	243,1	723,22
	644,63	269,08	378,94	719,68
	529,16	231,93	236,31	711,92
	594,4	313,3	117,02	713,92
	600,95	238,61	206,12	719,15
	413,29	262,59	247,87	733,99
	551,51	332,28	254,76	756,05
	546,62	345,78	163,48	772,83
	508,59	277,05	155,07	706,98
	419,87	268,6	191,91	732,59
	459,62	321,53	189,71	734,59
	418,17	196,35	76,26	716,81
	485,2	229,57	209,74	712,76
	441,75	318,98	225,43	704,89
	427,29	247,84	234,2	696,3
	468,98	288,37	207,09	699,85
	587,67	276,71	212,67	702,72
	554,47	314,58	248,38	702,86
	552,09	272,55	141,87	707,27
	559,83	302,38	250,06	708,1
	539,93	196,14	163,77	697,77
	531,56	149,2	255,91	695,36
	444,77	171,54	311,44	691,18
	489,79	207,51	141,92	1048,57
	402,61	177,33	188,26	1059,25
	426,15	316,66	244,82	1062,14
	549,54	284,36	179,97	1077,31
	556,62	329,66	162,04	1066,66
	559,3	370,32	272,04	1095,99
	578,08	265,56	219,61	1088,62
	520,82	296,59		1121,36
	560,1			1049,28
17	516,9869	269,2597	210,9923	816,5616

18	342,29	216,78	260,2	514,88
	357,72	329,02	204,54	460,54
	578,35	311,01	174,52	444,87
	336,77	188,79	208,74	476,54
	489,35	327,21	196,1	496,79
	443,05	213,3	237,35	476,69
	316,46	418,25	253,45	529,4
	583,49	209,21	165,45	533,15
	477,44	185,61	124,63	543,47
	474,23	344,79	101,55	540,22
	465,64	316,05	320,58	532,54
	529,97	310,05	210,66	535,54
	542,62	332,76	225,45	547,23
	500,6	297,8	179,89	548,35
	441,86	223,48	193,15	527,97
	349	223,58	143,43	519,5
	441,2	259,66	253,61	524,46
	492,32	295,48	246,31	520,54
	591,04	278,87	168,75	526,82
	561,11	321,28	158,42	550
	539,75	313,63	169,09	539,01
	514,81	292,91	249,24	561,03
	563,63	299,22	245,49	578,46
	592,15	278,14	332,01	581,78
	642,92	256,3	337,94	594,87
	621,85	208,59	359,82	580,49
	432,24	246,43	318,11	603,08
	450,17	300,22	296,06	696,24
	511,47	267,72	142,19	700,46
	485,56	298,09	135,14	677,4
	469,06	342,75	211,87	664,03
	541,49	249,28	170,02	650,4
	648,06	387,77	144,17	619,36
	614,69	300,78	157,25	574,45
	414,91	352,34	267,49	597,71
	460,66	294,05	191,43	558,19
	486,91	236,06	188,49	536,38
	360,56	202,63	226,74	557,47
	296,33	239,69		551,58
	370,02	292,67		
	440,61	189,59		
18	482,2527	279,3132	214,9824	558,2536

No			PE	
	Mucosa	Villi	Crypt	Muscularis
19	489,23	335,68	200,09	744,08
	640,69	496,49	114,62	742,63
	535,61	395,32	167,87	742,46
	576,22	361,64	285,64	745,7
	619,49	283	197,71	742,73
	760,21	277,9	205,42	733,94
	613,91	353,27	227,52	775,98
	617,18	218,42	145	790,14
	485,48	285,79	163,51	813,6
	441,88	305,84	139,6	813,86
	518,31	311,87	257,98	815,46
	482,83	297,23	208,83	823,82
	477,37	270,1	244,14	850,68
	529,44	329,76	208,74	851,81
	599,94	287,31	343	855,78
	672,23	264,88	169,49	864,34
	638,95	313,19	134,26	860,54
	569,35	312,18	187,77	883,58
	574,68	218,6	269,25	868,63
	690,93	235,14	136,86	867,52
	607,35	241,46	261,07	888,48
	582	292,93	266,92	854,82
	703,14	421,01	144,93	865,74
	610,48	299,25	325,12	873,58
	487,85	317,11	296,49	871,4
	492,88	357,38	283,47	882,15
	492,93	296,09	218,03	880,88
	583,75	317,64	222,16	897,04
	437,92	303,93	255,24	885,2
	620,02	166,14	275,01	884,4
	541,09	285,33	343,02	921,6
	559,64	282,8	187,15	919,64
	524,8	273,47	332,17	938,29
	537,72	219,11	340,36	938,19
	417,17	256,3	265,26	
	399,92	285,2		
	445,79	315,36		
	385,67	255,74		
	352,43	237,26		
	345,1	356,68		
	657,82	294,8		
	582,43	293,27		
	480,79	249,7		
	537,31	325,62		
	595,64	135,75		
	600,1	318,96		
19	545,9928	294,6065	229,2486	843,785

20	416,38	251,21	133,81	722,29
	384,12	208,53	246,03	735,59
	423,9	231,52	173,18	706,78
	358,77	161,04	188,47	706,09
	234,86	216,84	251,55	694,63
	386,31	298,01	301,65	680,66
	333,54	221,98	217,97	670,27
	409,31	271,49	146,06	666,5
	335,79	232,4	177,37	666,28
	485,31	252,99	117,18	665,5
	387,46	264,12	93,04	671,55
	426,88	157,25	110,46	654,77
	346,32	229,18	147,02	637,03
	343,16	213,26	226,44	631,34
	347,98	226,92	311,94	620,79
	443,25	188,36	193,92	615,88
	388,65	287,75	210,92	612,9
	453,01	231,17	246,81	611,9
	504,14	271,62	214,44	606,91
	438,26	297,12	236,06	606,07
	288,64	255,98	237,77	602,97
	455,87	235,16	264,39	589,79
	422,24	278,08	147,04	695,8
	388,64	339,84	125,46	670,6
	421,71	355,82	176,21	666
	387,25	363,99	136,58	663,84
	490,51	284,99	127,1	671,64
	578,43	318,65	143,42	669,9
	501,77	372,93	97,29	672,26
	419,81	302,54	213,91	635,27
	485,41	200,39	201,54	630,91
	475,92	252,84	196,38	631,66
	527,58	205		631,67
	448,37	253,17		
	431,75	303,84		
	422,26	193,6		
20	416,4878	256,3772	187,8566	655,0315

21	348,06	233,32	158,01	578,18
	342,76	279,29	269,15	597,37
	351,64	245,67	189,3	593,01
	387,22	194,54	170,9	597,37
	374,79	247,21	281,27	585,2
	333,41	219,5	161,86	589,51
	409,83	239,08	102,57	589,62
	335,06	247,86	181,29	589,56
	362,65	238,53	268,3	598,29

	411,26	232,94	195,54	592,07
	465,41	183,96	244,58	587,06
	353,42	272,37	309,44	589,2
	501,02	183,5	200,18	605,46
	498,53	254	237,07	578,57
	479,01	252,74	200,57	590,13
	397,31	220,79	205	597,86
	389,82	205,2	143,29	603,32
	384,22	210,55	142,74	611,38
	333,62	246,56	120,4	621,95
	391,59	261,28	242,92	629,54
	442,49	266,1	183,79	631,13
	471,06	289,07	168,88	1047,61
	507,91	203,15	194,01	1069,43
	451,69	208,12	228,49	1041,35
	370,58	244,2	147,97	1024,49
	377,41	225,35	81,2	1056,58
	416,87	221,63	154,81	1041,7
	442,91	316,76	172,78	1067,13
	412,09	289,01	205	1028,61
	379,91	226,48	178,51	1074,15
	384,34	269,96	107,36	1073,44
	406,65	273,29	165,73	1063,69
	400,88	343,36	169,21	1063,19
	432,27	280,3		1161,75
	491,36	304,11		1026,2
	551,61	266,18		
	572,75	295,05		
	541,57	196,01		
	333,9	183,38		
	294,02	385,01		
	381,47	390,81		
	384,13	344,58		
	450,84	358,1		
	466,63	230,31		
	554,16	219,01		
	475,23	228,84		
	462,75	202,02		
	398,52	236,59		
	467,17	263,55		
	512,67	231		
	393,15	215,07		
	395,11			
	406,65			
21	420,4034	252,4567	187,337	782,7171
22	362,63	209,37	176,63	854,11
	486,52	319,69	159,5	836,4
	394,76	237,55	132,18	851,05

	488,74	266,69	137,12	831,53
	475,78	263,74	306,63	819,31
	434,07	224,3	193,41	846,33
	378,74	173,1	258,18	818,2
	413,43	205,21	246,48	829,47
	401,31	245,73	190,98	876,31
	474,67	270,74	158,97	880,06
	647,94	390,47	208,03	891,76
	537,93	272,17	198,18	876,82
	649,37	295,32	324,13	862,66
	594,24	265,59	133,37	868,47
	612,3	320,35	186,91	858,17
	644,9	246,62	177,96	888,76
	651,66	254,37	233,25	922,55
	572,8	310,13	145,31	726,95
	541,35	226,77	241,07	716,91
	641,47	264,11	250,99	720,41
	658,75	262,97	260,09	729,86
	481,5	314,13	106,81	718,34
	442,26	263,07	120,59	735,98
	441,11	246,89	266,03	734,85
	463,78	290,87	251,18	904,97
	532,6	265,46	175,91	912,13
	477,2	265,03	183,41	921,44
	473,46	262,7	162,71	890,15
	371,11	223,48	176,62	848,12
	466,51	287,78	195,88	859,9
	627,45	343,6		862,51
	619,31	289,45		853,65
	490,57	254,79		835,97
	450,12	259,48		808,01
				789,49
				781,75
				767,43
				754,62
				747,97
				739,74
22	511,7747	267,4035	198,617	824,3278
23	386,22	231,6	203,74	544,95
	452,74	194,77	173,89	543,42
	396,53	217,11	202,09	551,88
	445,32	247,41	208,79	536,78
	527,83	188,14	191,54	522,53
	321,61	169,6	243,84	517,28
	479,53	251,6	195,62	521,43
	459,54	197,25	260,82	511
	517,58	204,97	271,92	509,7
	558,6	211,32	237,49	531,24

	594,18	213,31	277,55	537,47
	605,38	332,44	236,99	556,47
	539,14	371,52	229,64	558,32
	475,78	415,67	238,64	568,24
	469,86	328,13	163,77	581,73
	477,47	285,36	166,85	578,57
	417,04	212,14	187,09	567,68
	410,09	179,8	200,88	560,77
	393,55	220,63	218,04	553,49
	487,2	228,91	206,44	551,26
	445,25	247,24	300,84	709,85
	467,43	260,21	213,64	691,17
	430,88	195,46	217,65	682,81
	450,08	317,55	281,68	693,52
	361,09	243,73	254,65	711,06
	473,37	271,9	254,69	648,63
	570,12	241,37	284,84	668,34
	552,91	211,5	259,98	651,13
	442,28	318,18	219,73	663,68
	423,46	302,56	269,87	657,42
	471,84	236,81	237,45	658,55
	506,3	237,58	255,62	659,32
			219,57	660,33
			184,37	650,37
			239,71	676,38
			225,01	706,02
			189,23	758,89
			176,63	796,83
			133,97	
23	469,0688	249,5553	223,9682	611,8029
24	493,06	269,88	264,32	1171,58
	503,58	279,21	229,17	1229,57
	501,31	308,63	183,76	1344,17
	434,54	265,65	158,33	1243,13
	406,99	264,65	259,42	1202,83
	477,31	337,9	184,37	1147,6
	551,49	419,39	205,8	1129,65
	469	233,33	239,4	1098,94
	486,76	250,12	294,9	1041,43
	427,54	294,94	216,8	1347,42
	593,8	250,04	254,7	833,51
	515,28	226,1	212,76	823,47
	391,69	421,14	205,11	795,92
	408,86	306,57	277,08	766,26
	395,23	232,34	249,56	799,67
	451,42	263,3	372,21	783,68
	492,22	290,04	320,19	807,11
	496,36	241,77	290,04	789,89

	546,32	290,43	244,39	762,87
	462,7	261,28	246,92	700,77
	566,17	343,6	222,7	678,44
	606,87	334,12	211,36	711,5
	668,24	363,37	139,55	680,55
	563,41	267,52	116,16	681,26
	416,39	276,62	134,52	657,65
	487,1	253,27	183,21	627,6
	488,28	279,22	200,73	630,21
	524,06	279,01	209,4	632,19
	482,52	238,12	378,38	614,24
	491,72	331,72	208,14	636,67
				673,37
				684,01
				687,39
24	493,3407	289,1093	230,446	861,047
