# Supplementary material for: Effect of Replacing Soybean Meal by Raw or Extruded Pea Seeds on Growth Performance and Selected Physiological Parameters of the Ileum and Distal Colon of Pigs
Source: PLoS One. 2017 Jan 6;12(1):e0169467. doi: 10.1371/journal.pone.0169467 (PMC5218572; doi:10.1371/journal.pone.0169467)
Supplement: S3 Appendix — Raw data. (PDF) [file pone.0169467.s003.pdf]

S3 Appendix. Histology of the distal colon C75, raw data

No		C	
	Mucosa	Crypt	Muscularis
1	601,03	589,39	423,72
	606,41	585,98	444,52
	593,3	543,65	441,74
	631,95	522,73	419,94
	589,36	517,04	474,04
	626,89	462,74	496,17
	637,5	435,37	423,1
	630,1	524,82	475,33
	607,6	527,95	483,55
	662,2	488,13	468,79
	664,43	486,82	492,59
	625,45	451,02	532,79
	603,05	477,28	561,93
	602,27	491,78	587,51
	503,06	486,36	603,15
	513,09	509,37	614,95
	536,24	535,31	596,57
	528,07	564,9	597,22
	531,94	561,53	500,21
	553,25	600,29	484,09
	567,15	593,74	451,02
	549,65	526,21	431,48
	536,99	522,05	443,14
	529,17	517,57	450,74
	518,06	488,66	491,52
	487,51	481,14	497,65
	461,45	434,41	506,71
	490,08	540,77	514,02
	494,67	544,99	493,91
	526,8	546,65	475,39
	512,53	556,58	475,17
	481,08	522,24	470,27
	462,41	576,14	
	487,69	553,83	
	521,53	567,1	
	521,94	584,84	
	516,87	613,73	
	537,41	603,97	
	563,2	627,34	
	578,03	509,28	
	587,62	469,72	
	612,56	547,72	
	619,67		
	466,41		
	482,02		
	504,91		
	539,67		

	559,29		
	553,64		
	552		
	573,89		
	590,92		
	571,17		
	572,64		
	581,46		
	588,36		
	598,37		
	604,59		
1	557,769	530,7414	494,46656
2	601,64	601,01	517,03
	599,21	544,75	521,33
	621,04	526,64	539,93
	621,39	589,87	534,68
	611,61	621,98	552,68
	606,87	610,8	569,62
	603,01	592,54	569,21
	556,32	622,52	575,62
	645,29	480,44	528,54
	520,55	575,32	522,81
	658,8	572,15	553,4
	533,46	586,99	581,84
	537,45	537,83	572,09
	579,96	589,62	566,71
	606,57	586,06	576,02
	590,44	619,57	583,7
	637,88	614,92	588,73
	590,44	623,57	609,53
	610,9	543,06	572,79
	645,52	586,66	608,01
	655,76	577,43	621,19
	623,83	523,49	650,05
	635,07	528,54	659,87
	615,33	560,1	692,65
	628,84	619,56	695,71
	650,34	576,01	671,3
	635,48	622,8	661,18
	620,09	618,26	673,3
	518,82	619,9	618,32
	607,42	433	616,39
	577,11	574,16	605,13
	590,96	609,41	663,91
	686,55	618,1	
	612,71	545,8	
	635,25	606,15	
	629,31	542,35	

	646,59	582,27	
	623,29	588,08	
	616,43	591,73	
	627,26	578,24	
	626,2	640,03	
	642,83	545,54	
	657,25	570,07	
	633,86	636,27	
	634,34	555,95	
	637,75		
	636,55		
	654,56		
	633,01		
2	615,7376	579,7676	596,03969
3	446,46	436,04	605,58
	446,53	430,77	632,91
	475,66	441,07	508,31
	449,91	444,61	512,66
	452,24	415,37	516,22
	455,73	419,87	518,8
	436,14	396,97	532,79
	438,18	412,25	548,55
	435,47	405,19	562,44
	420,65	423,31	560,47
	416,78	357,44	573,71
	418,32	390,33	589,7
	421,85	400,78	618,53
	433,08	414,57	638,29
	419	392,51	514,1
	453,58	383,08	524,42
	445,79	367,56	532,61
	450,64	403,36	527,77
	435,88	433,92	517,51
	535,36	421,05	552,23
	526,64	473,45	530,06
	511,91	465,18	528,39
	502,08	503,38	504,51
	503,49	499,28	521,39
	515,26	495,16	513,28
	527,91	481,33	524,74
	513,79	429,22	503,72
	516,4	432,84	490,93
	514,28	447,39	530,11
	475,15	450,88	511,18
	450,42	418,27	528,85
	450,72	437,96	518,89
	480,88	411,36	
	463,66	422,24	

	457,67	423,6	
	465,35	440,08	
	433,16	381,99	
		492,35	
3	464,7573	428,8424	540,42656
4	713,26	670,39	621,77
	695,15	687,59	616,38
	695,72	700,28	607,57
	709,62	698,67	581,92
	712,36	669,36	593,9
	753,42	717,38	720,63
	757,52	690,36	779,28
	767,16	693,3	806,55
	771,16	731,81	903,12
	760,83	689,31	1071,78
	747,57	717,25	1057,51
	735,74	723,82	1031,65
	762,55	651,08	1029,77
	769,03	702	1020,74
	764,18	669,33	1038,46
	667,79	717,19	1040,4
	686,59	705,31	977,16
	717,91	726,43	1094,26
	719,73	724,67	892,57
	710,98	677,38	943,53
	722,54	702,78	728,02
	738,49	723,78	717,46
	710,67	631,37	688,79
	716,69	715,38	683,44
	720,02	727,73	639,07
	727,45	738,1	653,34
	749,12	730,69	654,27
	759,95	732,91	527,73
	773,19	720,09	703,98
	778,31	725,49	720,25
	710,62	640,34	
	691,5	736,39	
	757,99	685,67	
	791,92	666,05	
	744,97	705,48	
	742,78	716,5	
	760,33	705,31	
	765,64	713,67	
	749,49	692,5	
	731,39	676,61	
	738,39	651,41	
	733,1	678,13	
	764,42	695,4	

	752,56	710,26	
	750,8	709,13	
	736,79	693,66	
	755,27	684,38	
	719,58	703,06	
		679,71	
4	737,755	699,0794	804,84333
5	547,22	569,49	816,12
	560,51	515,32	828,33
	595,22	544,98	820,71
	604,46	584,09	780,3
	595,24	549,3	777,33
	601,74	561,49	695,31
	596,49	546,87	699,58
	600	554,03	666,35
	604,52	539,38	713,48
	613,04	545,92	868,78
	570,1	543,7	639,74
	568,99	552,96	678,55
	581,1	567,78	666,92
	600,92	596,36	621,5
	588,91	570,87	771,68
	576,73	591,9	724,97
	594,09	562,1	746,67
	599,24	570,62	705,19
	573,79	562,96	765,59
	571,21	565,08	821,59
	604,38	587,41	807,35
	599,03	582,77	897,69
	587,21	424,96	881,66
	594,25	583,42	851,71
	583,31	560,1	824,67
	595,76	531,94	731,13
	577,53	541,44	717,48
	567,94	582,11	
	604,47	540,14	
	585,59	576,26	
	598,03	504,86	
	576,43	540,28	
	610,83	554,88	
	606,83	543,14	
	606,2	562,27	
	603,63	570,44	
	604,43	585,99	
		572,51	
		568	
		575,44	
		577,75	

		595,24	
		603,59	
		536,16	
		548,84	
		565,14	
		417,96	
		609,83	
		571,87	
		560,37	
5	590,5235	557,4062	760,01407
6	592,8	578,64	658,3
	600,32	588,35	625,99
	591,26	583,39	623,91
	559,3	554,2	614,55
	579,79	540,33	609,78
	556,23	551,57	606,4
	575,45	578,44	616,72
	553,88	557,14	549,02
	568,45	568,72	560,12
	537,83	585,3	532,99
	535,89	542,82	569
	482,9	479,66	509,62
	522,58	532,29	555,49
	543,26	530,82	591,76
	638,27	527,08	544,93
	620,99	498,37	474,84
	590	585,62	496,19
	617,19	577,58	515,69
	644,8	560,84	520,39
	648,2	561,85	534,68
	574,11	545,88	544,53
	582,96	600,38	572,26
	578,09	611,77	518,76
	637,36	582,35	487,36
	651,53	559,64	444,19
	617,36	531,24	399,38
	619,37	556,73	497,42
	589,72	541,85	502,02
	577,1	560,56	495,25
	582,18	516,87	486,7
	634,15	585,46	508,65
	607,66	560,44	532,54
	603,53	583,29	553,64
	614,87	573,29	538,9
	624,46	577,93	532,04
	611,72		524,6
	561,99		500,41
	628,97		481,3

	621,85		450,68
			422,83
			419,97
			417,94
			452,23
			434,8
			410,27
			393,94
6	591,7531	559,1626	518,10826

No		PR	
	Mucosa	Cryp	Muscularis
13	529,12	501,71	479,87
	496,51	531,49	437,85
	448,38	496,15	439,09
	481,77	501,67	440,41
	511,76	499,01	465,8
	506,49	491,39	437,62
	454,91	425,32	447,25
	484,92	469,73	474,27
	474,33	553,33	397,55
	474,87	555,8	420,16
	484,73	550,95	418,11
	485,89	553,33	419,94
	497,12	558,27	405,77
	503,79	572,09	346,89
	503,14	541,21	341,71
	504,82	529,63	378,39
	516,75	516,47	386,93
	523,28	540,38	395,45
	527,87	510,4	394,07
	554,08	442,12	405,81
	546,69	463,47	386,93
	559,11	464,82	382,01
	574,68	461,82	384,63
	563,58	502,74	371,01
	570,2	467,11	383,08
	564,41	486,72	376,98
	559,68	486,24	379,91
	574,63	505,77	366,81
	563,8	506,03	356,68
	571,52	516,96	338,84
	554,79	478,51	350,55
	560,65	491,88	
		497,12	
		494,94	
		490,52	
		501,36	
		509,43	
		461,98	
13	522,7584	503,365	400,3345
14	595,38	530,52	548,27
	622,64	510,59	501,34
	616,85	516,79	509,24
	604,73	510,53	493,22
	595,5	480,85	509,78
	592,64	451,63	490,23

	581,35	390,28	470,01
	573,59	418,55	481,23
	573,59	509,68	434,38
	560,38	540,23	432,79
	544,16	524,75	450,57
	503,74	551,06	474,41
	465,09	561,65	443,28
	473,74	525,71	548,31
	455,02	509,7	530,49
	432,23	553,88	535,49
	568,28	558,94	484,34
	576,8	503,53	517,66
	572,39	491,67	591,65
	588,11	473,78	503,44
	587,93	502,84	494,75
	589,8	538,84	496,92
	589,14	594,93	483,32
	588,45	503,22	480,03
	591,95	499,23	498,05
	558,32	551,89	558,25
	524,7	512,29	604,06
	507,98	488,37	600,69
	531,7	547,62	622,52
	550,97	531,98	
	626,09	516,75	
	619,43	583,66	
	606,6	518,89	
	625,45	561,75	
	599,77	496,09	
	618,17	518,45	
	622,78	521,6	
	604,49	497,29	
	526,9	516,99	
	595,34	513,75	
	607,48	494,16	
	597,86	488,06	
	598,33	483,04	
	595,12	442,93	
	606,16	446,04	
	537,42	416,13	
		387,72	
		553,25	
14	571,8378	507,1267	509,9559
15	547,7	512,66	352,38
	561,59	510,08	334,31
	552,7	524,4	317,77
	529,05	529,39	374,95
	539,21	536,31	357,84

	561	503,09	396,04
	568,29	527,63	393,55
	544,5	525,25	422,98
	543,74	499,17	443,99
	539,34	525,13	427,01
	553,79	552,68	453,92
	544,97	519,85	443,78
	529,53	499,39	463,44
	548,81	529,25	454,09
	515,83	531,7	444,12
	539,05	519,83	440,37
	544,89	517,58	449,03
	540,44	527,44	408,34
	547,59	537,5	370,56
	561,03	514,88	374,88
	567,81	511,45	365,68
	551,79	533,21	342,28
	549,81	384,09	341,48
	542,95	444,69	352,95
	525,06	437,49	373,21
	527,6	447,41	368,19
	551,48	459,63	406,67
	544,88	487,82	397,3
	544,71	491,95	407,77
	554,26	457,78	457,37
			492,92
			460,14
			487,44
			474,33
			431,25
15	545,78	503,291	408,0666
16	616,96	501,7	554,03
	604,61	478,89	580,21
	611,5	487,1	565,37
	599,65	492,22	570,87
	571,69	510,7	578,45
	549,18	487,84	576,26
	489,88	548,65	555,4
	485,1	591,18	498,79
	501,6	587,94	512,2
	487,09	589,77	493,97
	517,27	561,92	557,87
	506,29	574,22	611,75
	493,21	489,29	618,44
	657,76	651,27	621,81
	685,12	647,79	576,05
	678,9	608,19	590,63
	673,39	617,07	631,25

	665,95	655,98	615,1
	662,02	634,14	620,38
	642,82	621,89	619,27
	655,9	644,6	631,49
	677,84	634,56	618,97
	624,26	645,34	648,92
	611,7	597,21	670,75
	626,19	658,12	695,7
	597,6	660,8	679,07
	549,44	640,03	695,96
	568,91	534,45	702,1
	556,44	437,24	689,61
	677,19	525,6	638,64
	676,69	630,25	673,17
	664,28	635,38	691,55
	645,16	487,02	657,42
	646,01	601,92	593,24
	654,71	503,5	646,32
	660,01	617,24	575,63
	651,91	627,97	
	630,63	651,67	
	661,55	603,15	
	635,21	628,95	
	650,37	639,38	
	655,73	630,4	
	679,12	606,34	
	669,53	616,38	
	609,83	613,89	
	644,1	592,47	
	647,5	636,83	
		635,8	
		606,75	
		644,44	
		672,39	
		668,34	
		620,79	
		588,75	
		618,79	
		631,61	
16	615,4851	595,1089	612,6844

17	551,61	500,25	478,19
	540,41	458,9	439,4
	543,4	528,23	427,24
	497,71	528,56	429,37
	562,34	508,29	407,65
	571,19	510,1	402,03
	571,99	543,77	478,74
	589,58	453,37	465,59

580,79	468,97	479,51
614,67	523,19	479,48
568,43	474,31	482,99
621,05	509,27	503,45
637,16	507,63	528,77
591,66	498,41	555,65
572,72	519,17	569,29
564,54	513,84	575,77
581,85	512,07	560,13
570,22	514,26	587
576,89	515,66	501,5
518,46		558,44
512,03		561,14
503,1		533,16
511,83		522,92
538,79		474,84
520,42		539,02
531,87		534,08
560,33		563,68
555,35		563,45
580,11		557,13
614,35		595,68
638,35		630,05
579,46		579,7
560,53		635,6
565,82		456,05
576,43		451,54
565,07		440,17
564,63		452,4
544,44		451,52
521,86		420,95
519,01		418,36
516,82		404,38
518,46		460,32
511		497,13
513,29		482,23
558,07		510,66
		505,81
		512,84
		521,81
		497,6
		473,75
		474,45
		477,44
		554,26
		560,64
		544,6
		548,3
		594,28
		650,65

			649,67
17	557,9576	504,6447	512,0754
18	674,79	632,95	556,89
	693,74	620,39	551,72
	648,81	609,77	601,8
	680,14	623,12	621,05
	679,15	648,85	635,88
	685,3	628,09	666,21
	670,09	662,17	689,38
	668,82	661,25	702,1
	679,83	650,75	673,99
	672,27	660,17	663,95
	690,86	680,38	625,56
	692,7	651,03	620,85
	687,64	634	596,02
	690,84	656,02	589,78
	680,1	648,4	530,59
	694,7	630,83	851,48
	694,87	609	858,37
	647,36	644,18	800,41
	677,4	606,79	771,68
	653,2	579,73	853,17
	672,36	595,43	826,49
	648,56	612,03	833,38
	653,09	650,73	784,73
	631,93	690,74	814,87
	654,69	692,41	880,9
	668,11	646,51	908,87
	648,12	616	892,54
	667,57	679,76	835,05
	654,77	683,95	840,41
	656,19	689,5	837,47
	676,52	677,18	921,08
	656,72	684,86	945,83
	650,79	658,71	941,96
	662,83	654,21	913,27
	696,29	678,72	866,92
	686,82		784,52
	690,48		
	711,13		
	709,96		
	689,66		
	683,15		
	687,99		
	654,26		
	702,17		
	668,62		
18	674,342	647,1031	758,0325

No		PE	
	Mucosa	Cryp	Muscularis
19	784,68	706,32	453,43
	768,63	728,98	451,33
	766,53	685,9	515,15
	752,69	688,5	535,96
	689,04	682,06	526,69
	714,61	674,55	511,91
	710,32	669,86	533,52
	718,77	652,05	502,61
	699,89	642,52	467,25
	657,64	651,51	470,7
	672,71	593,98	507,1
	662,97	651,47	512,88
	697,98	706,36	515,56
	736,42	719,68	514,56
	738,79	703,06	516,42
	719,34	704,75	533,52
	718,27	690,26	536,26
	725,55	687,55	465,41
	707,95	698,75	474,56
	696,01	684,62	460,54
	719,53	650,96	475,71
	701,47	730,27	508,61
	687,02	703,47	515,57
	699,95	693,19	444,92
	687,24	693,96	421,35
	729,66	724,6	422,29
	685,31	690,04	413
	707,91	712,26	408,78
	703,43	704,91	461,59
	731,76	642,87	504,4
	754,58	731,52	481,19
	722,18	738,39	461,98
	726,28	737,48	463,7
	740,74	736,19	440,56
	742,03	723,44	447,16
	725,68	711,53	496,15
	737,67	688,49	466,92
	744,67	689,71	469,01
	711,93	684,18	496,19
	674,31		424,3
	702,78		448,21
			480,25
			514,07
19	716,4615	692,569	480,7272
20	387,19	398,53	510,93

	391,07	380,18	550,48
	414,17	375,99	573,82
	416,31	377,55	546,56
	431,69	470,45	538,88
	447,43	522,74	505,39
	465,64	511,48	443,51
	480,73	534,68	480,58
	493,43	395,9	461,34
	495,98	379,35	393,31
	507,89	386,21	413,77
	510,25	369,51	411,4
	512,06	468,94	417,69
	509,18	530,31	463,88
	536,54	571,91	428,08
	569,49	406,47	659,58
	567,33	379,71	604,77
	575,73	380,05	487,87
	567,3	396,49	601,59
	566,34	456,49	593,65
	576,55	484,55	633,37
	599,74	440,17	647,34
	585,15	504,71	631,34
	589,65	507,55	600,71
	615,34	512,93	490,62
	608,04	536,26	445,66
	605,67	540,33	437,78
	614,69	525,59	449,96
	463,46	536,67	443,43
	520,32	521,83	440,61
	584,88	536,79	413,55
	558,15	501,31	557,04
	596,9	380,31	413,82
		386,12	421,17
			474
			456,41
20	526,1906	459,0606	501,2192
21	505,19	437,12	879,2
	480,48	467,54	906,47
	461,34	488,77	940,62
	444,89	498,43	999,55
	450,13	508,28	995,69
	472,56	491,72	974,47
	465,25	502,4	984,4
	455,3	491,95	974,46
	459,71	483,12	963,22
	494,12	535,69	953,38
	509,03	501,94	875,76
	507,64	538,1	885,64

	497,4	526,64	894,24
	502,81	530,15	937,31
	497,53	518,88	884,36
	493,9	497,4	961,42
	503,92	500,66	968,28
	538,43	488,66	1099,76
	540,14	444,84	1132,61
	477,75	480,95	1122,12
	501,62	514,52	1091,48
	520,52	516,64	1131,02
	501,64	496,67	1016,69
	514,44	497,13	902,64
	543,8	538,83	882,13
	542,62	511,18	734,15
	537,65	525,28	777,73
	530,81	512,68	1143,48
	550,51	528,07	866,29
	522,81	501,12	770,39
	520,32	499,21	762,59
	530,82	494,97	
	531,13	529,5	
	520,59	523,57	
	502,2	522,5	
	520,94	544,53	
	525,23	530,67	
	522,81	519,61	
	537,84	522,81	
	493,43	476,6	
	532,63	496,34	
	544,41		
	538,06		
	545,94		
	538,95		
	537,49		
	531,88		
	551,29		
	544,25		
	562,43		
	532,78		
	516,53		
	491,41		
	529,35		
21	513,4565	505,748	948,7597
22	711,64	668,01	581,78
	698,64	646,03	561,45
	678,89	623,56	519,39
	672,54	657,99	520,9
	652,81	658,69	543,95

	680,6	628,05	558,62
	688,7	640,05	581,12
	681,26	629,29	454,63
	678,03	636,07	473,29
	670,68	645,16	484,42
	662,83	612,84	550,79
	676,02	650,44	507,7
	684,55	684,87	506,75
	667,29	672,55	513,41
	677,63	644,08	495,93
	648,03	645,67	476,36
	650,44	665,11	465,56
	645,11	660,45	475,29
	622,37	638,28	465,25
	634,96	649,6	495,69
	639,36	632,42	484,97
	654,06	629,8	492,42
	656,17	647,11	465,17
	665,28	673,74	483,08
	637,56	580,69	490,46
	640,45	611,6	480,51
	654,68	622,46	481,17
	633,58	614,56	507,66
	636,83	597,23	551,79
	637,43	621,47	549,33
	629,25		534,53
	638,87		533,25
	663,42		509,56
	702,03		509,5
	707,87		543,45
	720,62		592,67
	725,64		
	694,74		
	691,39		
	687,2		
	683,07		
	685,79		
	674,98		
	664,32		
	673,66		
	659,11		
	673,98		
	700,26		
	692,37		
22	669,5304	639,5957	512,2722
23	642,11	623,53	545,03
	642,3	671,28	561,07
	655,28	646,26	567,51

	658,19	570,07	560,27
	622,46	635,7	573,65
	605,06	614,46	537,17
	646,4	619,76	518,77
	652,93	585,82	516,24
	664,05	552,36	510,73
	670,97	595,01	513,61
	637,78	645,3	551,49
	640,78	654,71	574,35
	634,67	686,68	560,87
	634,62	706,73	502,25
	630,27	713,25	482,66
	632,59	677,61	534,26
	605,7	663,11	537,41
	653,51	668,29	551,63
	688,55	679,59	546,75
	782,76	707,16	548,97
	751,45	730,5	542,41
	786,08	677,83	527,41
	750,23	657,12	530,12
	771,48	647,87	541,21
	709,68	632,65	530,12
	702,27	636,81	551,26
	707,63	606,8	594,77
	733,53	607,85	596,39
	700,12	628,98	568,1
	679,07	665,78	551,5
	739,81		550,29
	750,08		553,79
	738,14		
	714,56		
23	683,3856	646,9623	544,7519
24	568,1	677,83	495,28
	551,5	657,12	489,99
	550,29	647,87	472,56
	553,79	632,65	478,06
	553,68	636,81	499,24
	540,8	606,8	473,49
	518,58	607,85	499,39
	514,05	628,98	558,14
	553,91	571,58	571,75
	576,11	580,84	577,78
	583,67	542,99	576,85
	569,79	569,03	552,8
	575,35	520,94	554,7
	595,14	492,6	614,58
	572,76	509,56	684,86
	561,46	581,53	782,33

	573,33	566,97	828,41
	572,38	505,11	447,39
	596,26	528,64	452,06
	575,73	562,66	464,28
	591,66	538,94	467,16
	614,96	505,86	471,56
	586,13	560,86	475,76
	582,87	550,06	440,25
	551,54	581,53	474,08
	594,25	512,46	477,46
	595,49	526,95	447,96
	605,83	588,26	448,24
	616,14	582,73	414,56
	586,82	581,1	427,53
	609,81	589,76	
	591,66	590,76	
	606,53	575,47	
	596,78	555,05	
	611,83	612,74	
	611,6	633,53	
		522,6	
		473,78	
24	578,0717	571,3368	520,6167
