# Supplementary material for: Effect of Replacing Soybean Meal by Raw or Extruded Pea Seeds on Growth Performance and Selected Physiological Parameters of the Ileum and Distal Colon of Pigs
Source: PLoS One. 2017 Jan 6;12(1):e0169467. doi: 10.1371/journal.pone.0169467 (PMC5218572; doi:10.1371/journal.pone.0169467)
Supplement: S4 Appendix — Raw data. (PDF) [file pone.0169467.s004.pdf]

S4 Appendix. pH and short chain fatty acids concentration, raw data.

	acetate	propionate	isobutyrate	butyrate	isovalerate	valerate	total		pH
C	29,21	12,79	0,98	8,46	1,34	1,15	53,93	C	6,43
C	39,25	19,06	1,46	11,05	2,43	1,48	74,72	C	6,53
C	21,89	6,97	0,86	3,67	1,11	0,68	35,18	C	7,28
C	30,78	8,61	1,42	8,73	1,94	1,23	52,72	C	7,01
C	36,13	10,33	0,96	7,76	1,14	2,06	58,39	C	6,76
C	35,09	9,81	1,06	7,78	1,32	1,33	56,40	C	6,87
PR	20,96	8,15	0,77	5,27	0,99	0,59	36,73	PR	7,11
PR	29,59	14,11	1,37	10,19	2,06	1,36	58,69	PR	6,75
PR	24,32	10,89	0,79	12,28	1,02	1,82	51,12	PR	6,48
PR	35,52	11,78	1,06	11,45	1,38	1,17	62,38	PR	6,4
PR	31,37	13,74	0,92	12,10	1,12	1,33	60,58	PR	6,56
PR	38,55	15,18	1,06	13,95	1,63	1,53	71,90	PR	6,14
PE	28,97	12,01	1,19	5,75	1,60	1,21	50,73	PE	6,91
PE	32,66	14,19	1,62	8,98	2,52	2,62	62,59	PE	6,87
PE	32,85	9,63	1,61	6,94	2,35	1,35	54,72	PE	7,06
PE	25,91	9,72	1,11	4,57	1,46	0,94	43,71	PE	6,99
PE	30,32	8,76	1,08	4,95	1,37	0,73	47,21	PE	7,08
PE	25,13	9,89	1,48	5,66	2,07	1,03	45,26	PE	7,12
