# Supplementary material for: Effect of Replacing Soybean Meal by Raw or Extruded Pea Seeds on Growth Performance and Selected Physiological Parameters of the Ileum and Distal Colon of Pigs
Source: PLoS One. 2017 Jan 6;12(1):e0169467. doi: 10.1371/journal.pone.0169467 (PMC5218572; doi:10.1371/journal.pone.0169467)
Supplement: S5 Appendix — Raw data. (PDF) [file pone.0169467.s005.pdf]

S5 Appendix. Bacterial enzymes activity, raw data.

	β-glucosidase		β-glucuronidase
C	65,63	C	13,70
C	182,47	C	39,26
C	57,61	C	14,38
C	99,04	C	63,07
C	68,98	C	15,68
C	62,20	C	34,85
PR	50,98	PR	9,56
PR	65,39	PR	5,77
PR	52,46	PR	13,44
PR	92,34	PR	20,06
PR	138,61	PR	14,14
PR	60,51	PR	36,16
PE	86,69	PE	14,45
PE	110,94	PE	31,06
PE	53,86	PE	17,04
PE	100,10	PE	20,99
PE	66,77	PE	30,43
PE	130,60	PE	39,95
