# Supplementary material for: Effect of Replacing Soybean Meal by Raw or Extruded Pea Seeds on Growth Performance and Selected Physiological Parameters of the Ileum and Distal Colon of Pigs
Source: PLoS One. 2017 Jan 6;12(1):e0169467. doi: 10.1371/journal.pone.0169467 (PMC5218572; doi:10.1371/journal.pone.0169467)
Supplement: S6 Appendix — Raw data. (PDF) [file pone.0169467.s006.pdf]

S6 Appendix. PCR, raw data.

| | *Lactobacillus* spp. (286 pz) | *E. coli* (584 pz) | *Clostridium* spp. (722 pz) | *Bifidobacterium* spp. (1420 pz) |
|---|---|---|---|---|
| C | 0,052501073 | 0 | 0,2251044 | 0,132987879 |
| C | 0 | 0 | 0,5748394 | 0 |
| C | 0,215689834 | 0 | 0,4175513 | 0,363141275 |
| C | 0,717371041 | 0 | 0,5684083 | 0 |
| C | 0,244967016 | 0 | 0,8194744 | 0 |
| C | 0,021767615 | 0 | 0,5112341 | 0 |
| PR | 1,016960461 | 0 | 0,5647527 | 0 |
| PR | 0,026684807 | 0,016362991 | 0,5973563 | 0,756268012 |
| PR | 1,079197273 | 0 | 0,5784765 | 0 |
| PR | 0,417775861 | 0,012522141 | 0,5229361 | 0 |
| PR | 0,058273412 | 0 | 0,5747917 | 0,356207461 |
| PR | 0,318166085 | 0 | 0,6745005 | 0 |
| PE | 0,668527375 | 0 | 0,6449215 | 0 |
| PE | 0,063823057 | 0 | 0,7238525 | 1,046100478 |
| PE | 0 | 0 | 0,7452057 | 0 |
| PE | 0,192005444 | 0 | 0,8291672 | 0,084526161 |
| PE | 0,185645131 | 0 | 0,5555799 | 0 |
| PE | 0 | 0 | 0,773943 | 0,89810593 |
