# Supplementary material for: Effect of Replacing Soybean Meal by Raw or Extruded Pea Seeds on Growth Performance and Selected Physiological Parameters of the Ileum and Distal Colon of Pigs
Source: PLoS One. 2017 Jan 6;12(1):e0169467. doi: 10.1371/journal.pone.0169467 (PMC5218572; doi:10.1371/journal.pone.0169467)
Supplement: S7 Appendix — Raw data. (PDF) [file pone.0169467.s007.pdf]

S7 Appendix, Amines concentration, raw data.

	spermidine	methylamine	putrescine	phenylethylamine	cadaverine	Histamine
C	0,0179	0,0178	0,0277	0,0074	0,0115	0,0210
C	0,0180	0,0203	0,0278	0,0102	0,0090	0,0190
C	0,0178	0,0143	0,0596	0,0095	0,0098	0,0200
C	0,0110	0,0179	0,0264	0,0071	0,1033	0,0186
C	0,0086	0,0169	0,0163	0,0062	0,0101	0,0190
C	0,0179	0,0173	0,0326	0,0075	0,0143	0,0199
PR	0,0148	0,0127	0,0561	0,0081	0,0184	0,0194
PR	0,0173	0,0168	0,0292	0,0073	0,0166	0,0189
PR	0,0121	0,0148	0,0155		0,0973	0,0192
PR	0,0183	0,0168	0,0233	0,0085	0,0081	0,0190
PR	0,0152	0,0188	0,0264	0,0073	0,0156	0,0179
PR	0,0218	0,0178	0,0434	0,0122	0,0095	0,0184
PE	0,0166	0,0167	0,0221	0,0086	0,0094	0,0205
PE	0,0368	0,0172	0,0372	0,0112	0,0357	0,0263
PE	0,0153	0,0166	0,0155	0,0068	0,0087	0,0181
PE	0,0226	0,0162	0,0366	0,0081	0,0131	0,0219
PE	0,0154	0,0170	0,0278	0,0102	0,0783	0,0206
PE	0,0306	0,0206	0,0251	0,0111	0,0899	0,0180

1,7diaminoheptane	tyramin	tryptamine	total
0,0362	0,0058	0,0166	0,1619
0,0595	0,0045	0,0204	0,1886
0,0465	0,0022	0,0171	0,1968
0,0488	0,0060	0,0139	0,2531
0,0375	0,0194	0,0124	0,1464
0,0394	0,0093	0,0182	0,1764
0,0206	0,0076	0,0142	0,1720
0,0417	0,0049	0,0219	0,1745
0,0325	0,0053	0,0000	0,1966
0,0545	0,0040	0,0120	0,1643
0,0383	0,0037	0,0000	0,1432
0,0529	0,0044	0,0205	0,2008
0,0572	0,0048	0,0116	0,1675
0,0624	0,0122	0,0236	0,2625
0,0575	0,0038	0,0141	0,1565
0,0443	0,0094	0,0263	0,1985
0,0477	0,0053	0,0218	0,2442
0,0599	0,0153	0,0226	0,2932
